# Borromean three-body FRET in frozen Rydberg gases

**DOI:** 10.1038/ncomms9173

**Published:** 2015-09-08

**Authors:** R. Faoro, B. Pelle, A. Zuliani, P. Cheinet, E. Arimondo, P. Pillet

**Affiliations:** 1 Laboratoire Aimé Cotton, CNRS, Univ. Paris-Sud, ENS Cachan, Bât. 505, 91405 Orsay, France; 2 Physics Department, Universita di Pisa, Largo Pontecorvo 3, I-56127 Pisa, Italy; 3 INO-CNR, Via G. Moruzzi 1, 56124 Pisa, Italy

## Abstract

Controlling the interactions between ultracold atoms is crucial for quantum simulation and computation purposes. Highly excited Rydberg atoms are considered in this prospect for their strong and controllable interactions known in the dipole-dipole case to induce non-radiative energy transfers between atom pairs, similarly to fluorescence resonance energy transfer (FRET) in biological systems. Here we predict few-body FRET processes in Rydberg atoms and observe the first three-body resonance energy transfer in cold Rydberg atoms using cold caesium atoms. In these resonances, additional relay atoms carry away an energy excess preventing the two-body resonance, leading thus to a Borromean type of energy transfer. These few-body processes present strong similarities with multistep FRET between chromophores sometimes called donor-bridge-acceptor or superexchange. Most importantly, they generalize to any Rydberg atom and could lead to new implementations of few-body quantum gates or entanglement.

## Abstract (web summary)

Rydberg atoms are promising platform for quantum simulations, due to their strong and controllable dipole–dipole interactions. Here, the authors predict few-body processes in Rydberg atoms which resemble fluorescence resonance energy transfer in biological setting, and observe them in cold caesium atoms.

## Graphical abstract

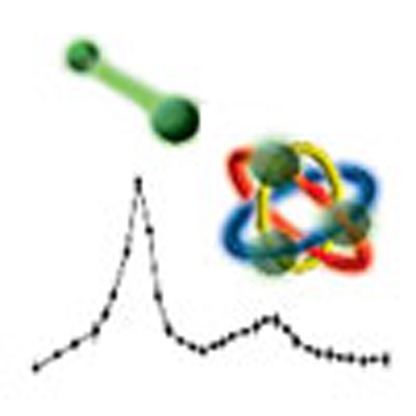

Ultracold atom experiments are recognized as promising for quantum simulation^1^,^2^ and have gathered important achievements, reproducing many-body Hamiltonians as for instance the Superfluid to Mott insulator transition^3^, the Bose-Einstein Condensate to Bardeen-Cooper-Schrieffer (BEC-BCS) crossover^4^ and the Tonks–Girardeau gas^5^,^6^ or realizing complex one-body Hamiltonian as the quantum transport problem of Anderson localization in three dimensions^7^,^8^ for which no exact solution is known.

In most of these experiments, the atomic interaction is a key parameter and its control, when possible, is a valuable asset. Highly excited atoms, usually called Rydberg atoms, display strong tunable interactions and have thus been proposed for both quantum simulation^9^ and quantum computation^10^,^11^ using the interaction blockade^12^ induced by their van der Waals interactions scaling typically as *n*^11^ with *n* the principal quantum number. This approach has already proven successful, exploiting the Rydberg blockade^13^,^14^ for the consecutive creation of entanglement^15^ and implementation of a quantum gate^16^. More recently, Rydberg–Rydberg interactions have been used to apply a phase shift in an atomic interferometer^17^. These results are demonstrating Rydberg atoms potential.

The van der Waals interactions arise from out-of-resonance dipole–dipole interactions, which can be tuned to resonance under the proper electric field^18^ thanks to the Stark effect. When this condition is fulfilled, they can also lead to non-radiative energy transfers called Förster resonances due to their similarity with fluorescence resonance energy transfer^19^ (FRET) that has been presented by Förster^20^ to explain energy transport in biological systems, after a purely quantum description by Perrin^21^. The theoretical coherent nature of FRET has been recently demonstrated in Rydberg atoms^22^ thanks to the precise control of atomic positions in this experiment.

The similarity between Rydberg physics and FRET in biological systems has motivated the use of Rydberg atoms for the study of quantum energy transport^23^,^24^ induced by two-body FRET in many-body systems. In parallel, biological studies are trying to determine to what extent the quantum nature of FRET remains at ambient temperature^25^ and how few-body mechanisms might influence the energy transport: multistep FRET^26^ has been demonstrated using relay chromophores or quantum dots, even for non-resonant relays^27^ in so-called donor-bridge-acceptor or superexchange configurations^28^.

Similarly, few-body interactions have recently found a renewed interest in ultracold atomic physics community as paving the way between two-body and many-body physics, as for Efimov physics^29^,^30^, three-body recombination^31^, macro-molecule formation as Rydberg macro-trimers^32^,^33^ and few-body interactions^34^, with a special interest to the so-called Borromean interactions displaying strong three-body interactions with a negligible contribution of two-body interactions^33^,^35^. Nevertheless, these studies remained up to now theoretical in Rydberg physics where no Borromean interaction could be demonstrated.

The present work predicts new few-body FRET processes in Rydberg atoms and presents the first observation of a three-body FRET in a cold Rydberg gas. It corresponds to a generalization of the usual two-body FRET where a third atom serves as a relay for the energy transport. This relay also compensates for the energy mismatch preventing the direct two-body FRET between the donor and the acceptor which thus results in a Borromean three-body energy transport. The predicted few-body FRET can be generalized for any quantum system displaying two-body FRET from quasi-degenerate levels. It thus promises important applications in the formation of macro-trimers, implementation of few-body quantum gates, few-body entanglement or heralded entanglement.

## Results

### Two-body resonant interaction

In cold Rydberg atoms physics, FRET resonance condition is usually achieved applying an electric field *F* that Stark shifts the involved energy levels^18^, enabling to find a large set of resonances. In caesium^36^ a well known two-body FRET is described by the equation:


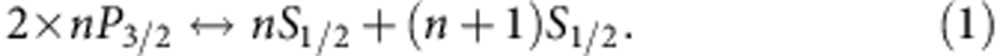


where *n* is the principle quantum number, *S* (respectively *P*) represents the orbital angular momentum 0 (resp. 1) of the excited electron and the subscript specifies the total angular momentum *J*. We notice that this resonance exists for both total angular momentum projections |*m*_*J*_|=1/2 and |*m*_*J*_|=3/2 of an initial *nP*_3/2_ state or for a mixture of both. For convenience, the Rydberg states *nS*_1/2_, (*n*+1)*S*_1/2_, *nP*_3/2_|*m*_*J*_|=1/2 and *nP*_3/2_|*m*_*J*_|=3/2 will now be labelled as *s*, 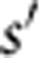, *p* and 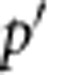, respectively, with respective energies *E*_s_, 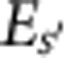, *E*_*p*_ and 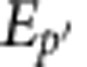 which all depend on the applied electric field *F*. It is important to notice that the zero-field *p* and 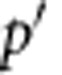 degeneracy is lifted around the resonant FRET electric field, providing a small but finite energy difference that we define as 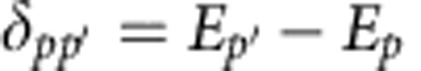 on the order of several tens of MHz. As this is much smaller than the energy difference of around 10 GHz between *s*, 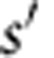 and *p* or 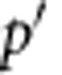, equation (1) represents an energy transfer between an initial species (*p* states) and a different one (*s* states).

**Figure 1:**
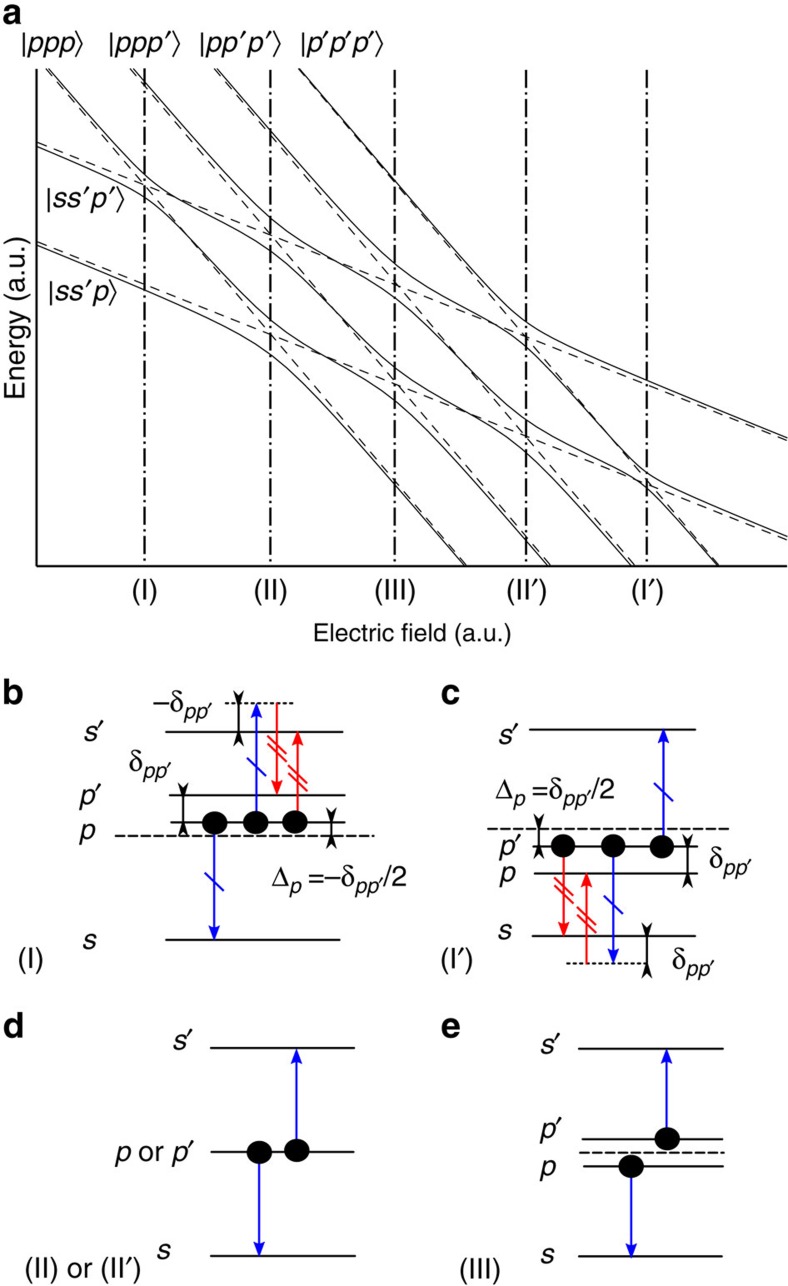
Two-body and three-body FRET processes. (**a**) Schematic energy diagram as a function of the applied electric field for the different three-atom states involved: the dotted lines represent the energy of each state in the abscence of interactions, for example, for atoms far apart. Interactions turn the different resonances into avoided crossings marked with dash-dotted lines. They correspond to two-body resonances ((II), (III) and (II′)) and three-body resonances ((I) and (I′)) where the resonant energy transfers detailed in (**b**) to (**e**) can take place. (**d**) Describes how the two-body FRET (II) (resp. (II')) from the *p* (resp. 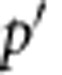) state occurs through the exchange of a single virtual photon symbolized with two blue arrows. (**e**) Describes the similar two-body FRET from a *p* and 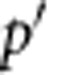 mixture. (**b**) (resp. (**c**)) Describes how the three-body FRET transfers the starting *ppp* (resp. 
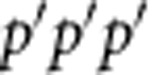
) state to the end state through the exchange of two different virtual photons. The first virtual photon, symbolized with blue arrows crossed with a single mark, leads the system to a virtual intermediate state before the second, symbolized with red arrows crossed with two marks, leads it resonantly to the end state. For reference, an horizontal dashed line at the energy 
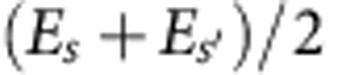
 represents when possible the two-body resonance condition.

We introduce the one atom energy mismatch Δ between the starting state and the average energy of the end state, so-called Förster defect. For the two starting states *p* and 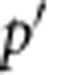 we define 
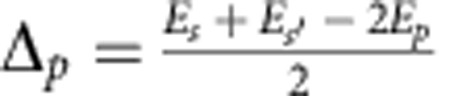
 and 
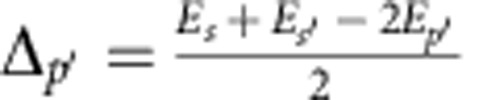
. The different two-body FRET resonances are associated to an initial couple of *p* states (II label), initial 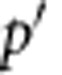 states (II' label), and finally to an initial 
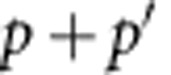
 couple (III label) with respective resonance conditions Δ_*p*_=0, 
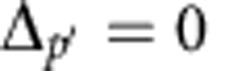
 and 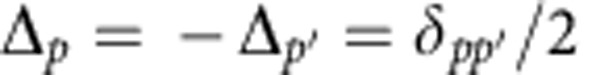. The schematic three-body energy diagram of Fig. 1a shows how the three possible two-body FRET resonances arrange as a function of the electric field in six avoided crossings arranged by pairs of the initial states. The bottom diagrams of Fig. 1b represent the different two-body FRET in a two-particle picture, omitting the third inactive atom in the three-particle picture of Fig. 1a.

In caesium these resonances can be explored for all *n*<42 using the Förster defect electric field dependence^36^. For each resonance, we consider the angle-averaged dipole–dipole interaction coupling term *V*_dip_ which is proportionnal to the two transition dipole moments *μ*_*ps*_ and 
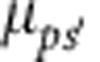
 between *p* and *s* (resp. 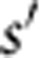) and inversely proportional to the cube of the interatomic distance *R* such that 
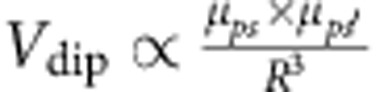
. The *n*^2^ dependence of the dipole moments in Rydberg atoms implies thus a *n*^4^ dependence of the dipole–dipole interaction.

### Three-body resonant interaction

Considering now a third atom, we introduce two non trivial novel FRET processes starting from *p* or 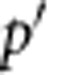 states as described by the following equations:


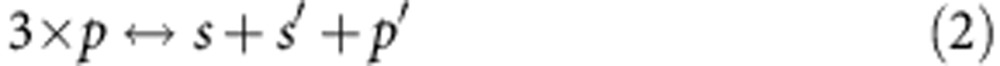



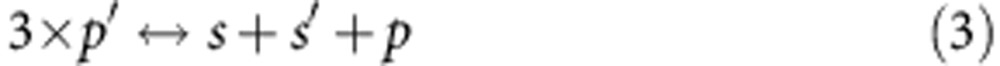


Both processes are three-body coherent FRET processes and correspond to the two outer avoided crossings, (I) from *p* and 
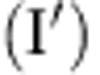
 from 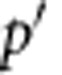 in Fig. 1a. They appear at specific electric fields well separated from the two-body FRET due to the finite energy 
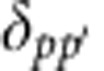
. The three-body resonance conditions are respectively 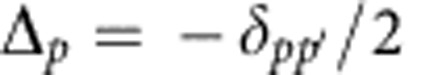 and 
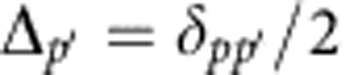
. In the above three-body processes, one particle changes only weakly its energy and can be seen as assisting the two-body FRET at a different resonance condition. This atom will relay the energy between the two other atoms.

The top diagrams of Fig. 1b represent the three-body (I) (resp. 
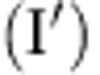
) FRET where the centre atom, transferred to 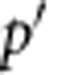 (resp. *p*), acts as the relay contributing twice to the whole energy transfer. In this figure this is displayed by the two arrows acting on the relay atom. The specificity of these processes resides in the absence of a real intermediate state, which is detuned by 
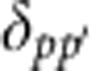
. Despite the use of a relay, the transfer occurs in a single step, implying a Borromean character of the relay atom which absorbs the energy of the finite Förster defect. Indeed at the three-body resonant field the two-body FRET is forbidden and only the single three-body resonance is allowed. The overall process corresponds to the transfer of one excitation over the three atoms. When 
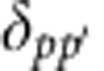
 is large compared to the dipole–dipole interactions, the three-body coupling can be perturbatively evaluated to a coherent interaction with the coupling 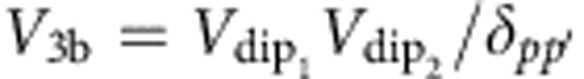 where 
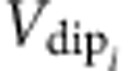
 is the dipole–dipole interaction coupling the Borromean atom to the *i*th other atom.

### Experimental protocol summary

In our experimental set-up described in ref. 34, the preparation in an *nP*_3/2_ Rydberg state (with *n* between 28 and 35) is produced through a three-photon resonant excitation using two intermediate states 6*P*_3/2_ and 7*S*_1/2_. The short lifetime of these intermediate states of around 30 and 50 ns, together with the duration of the excitation pulse of typically 200 ns, broadens the excitation linewidth to around 10 MHz. It limits strongly the excitation blockade, leading to high Rydberg atom densities with random distributions. The random interatomic distances and orientations in the Rydberg sample justify the use of the angle-averaged interaction at the average distance in our calculations and will prevent the observation of coherence properties. We then let the system evolve for times 
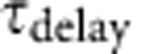
 ranging from 300 ns to 1 μs during which the energy transfer takes place towards a final population in *s* and 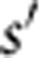 states. Finally we apply an ionization ramp to obtain a time-of-flight (TOF) signal and extract the populations *N*_*j*_ in each Rydberg state *j*, appearing in the TOF in the order 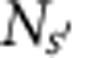, *N*_*p*_, 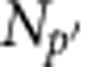 and finally *N*_*s*_. We will usually express the results in terms of the population transfer ratio 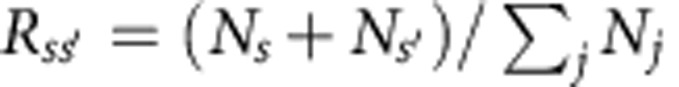 describing the fraction of Rydberg atoms transfered to the *s* or 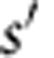 states. The population detection presents cross-talks on the order of 10–20% due to avoided crossings in the ionization path but we correct for them using a simple matrix algorithm already presented in ref. 34. This correction requires taking a reference TOF for each considered state at a reference electric field *F*_0_ away from any resonances. The final uncertainty in population transfer ratios 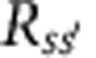 is evaluated to be around ±0.5% for data at electric fields on the same side of the main two-body FRET resonance than *F*_0_. On the other side, larger residual cross-talk errors up to 5% for *n*=35 can persist due to the interactions during the ionization ramp. We therefore always paid attention to take the reference TOF on the same side as the expected three-body FRET. After the initial *p* or 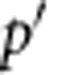 preparation, the tuning of the electric field gives access to resonances (I) and (II) (resp. 
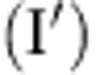
 and 
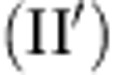
), but never to resonance (III) which requires a *p* and 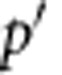 mixture.

### Resonances

**Figure 2:**
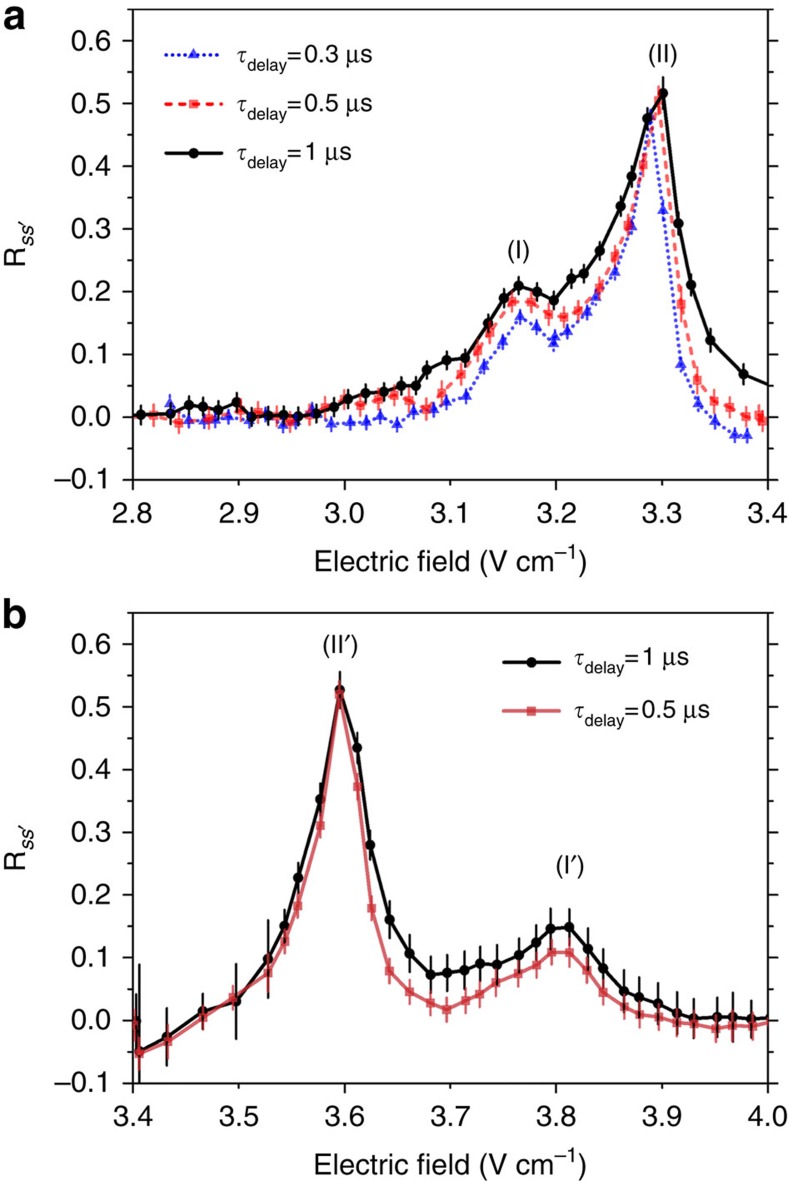
Population transfer. Averaged transfer ratio 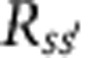 from the initial *p* state (**a**) or 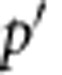 state (**b**) to 
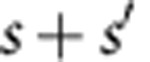
 states for *n*=35 at different 
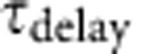
 versus the electric field. In (**a**), the resonance at around 3.3 V cm^−1^ is attributed to the two-body FRET (II) while the resonance at around 3.17 V cm^−1^ is attributed to the three-body FRET (I). In (**b**), the resonances are attributed to (II') and (I'). Each point corresponds to the average of 50–150 individual measurements. The error bars correspond to the sum of the s. e. m. and the estimated error in the state discrimination.

Figure 2 reports the results for *n*=35, which leads to the largest measured transfer due to the *n*^4^ dependence of *V*_dip_ and to the smallest 
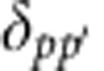
.

In Fig. 2a, we plot the population transfer ratio versus the applied electric field *F* and display the result from the *p* state for different transfer delays 
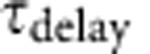
. We resolve two peaks corresponding to the two-body (II) and three-body (I) FRET at 3.30 V cm^−1^ and 3.17 V cm^−1^, respectively. They are in good agreement with the values of 3.28 V cm^−1^ and 3.15 V cm^−1^, calculated using the numerical method proposed in ref. 37, with our uncertainty on the applied electric field of ±0.02 V cm^−1^ and on the calculated resonance position of ±0.05 V cm^−1^. The observed two-body transfer saturation limit of 50% is due to the random distribution of Rydberg atoms in our sample leading to a statistical average between the two-body states |*pp*〉 and 
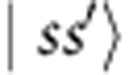
. We observe an efficient three-body FRET transfer of up to ∼21% close to, but below, the expected maximum of 33% for a statistical average between |*ppp*〉 and 
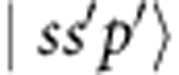
.

Figure 2b presents the analogous results from the initial 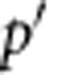 state, corresponding to the three-body FRET of equation (3). The resonance fields are again in excellent agreement with the predicted values of 3.60 V cm^−1^ and 3.80 V cm^−1^ for (II') and (I'), respectively. In addition, we see that the two peaks are better resolved due to the increase of the energy splitting 
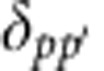
 with the electric field. It also implies a smaller three-body coupling *V*_3b_ and the observed transfer efficiency is smaller with around 15% transfer.

The application of different delays in Fig. 2 enables us to observe the transfer time evolution. At our experimental density of ∼5 × 10^9^ atoms cm^−3^, the typical two-body FRET coupling *V*_dip_ is expected to reach a maximum of 15 MHz at the average interatomic distance of 3 μm and the transfer is fully saturated at the shortest delays. This interaction level, larger than the excitation linewidth, is large enough to induce a partial excitation blockade effect and could induce the direct excitation of one of the two-body exciton states 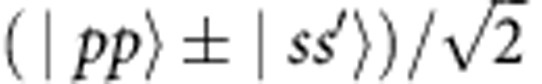 still corresponding to the 50% expected saturation transfer. On the contrary, with 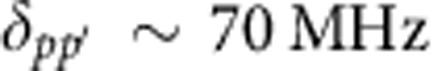 for FRET (I) (resp. 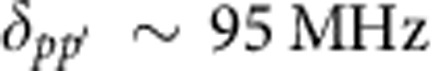 for FRET (I')), the three-body interaction coupling *V*_3b_ is expected around 3 MHz (resp. 2 MHz) at 3-μm interatomic distances. As a consequence, we observe a time evolution at the shortest delays. Unfortunately, the strong dependence of the three-body interaction *V*_3b_ with the interatomic distances and the random distribution of the Rydberg atoms in our sample prevent us from observing any oscillations in the energy transfer.

### Density dependence

To further demonstrate the three-body character of this phenomenon, we studied the transfer dependence on the initial Rydberg atom density using the technique developed in ref. 34. Indeed in the low density limit, a N-body process will depend as the N-power of the initial density. We have chosen the 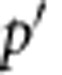 starting state and a transfer time of 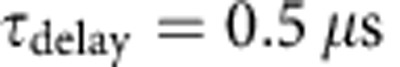 where the resonances are better resolved and the signal on one resonance is less impacted by the other resonance.

**Figure 3:**
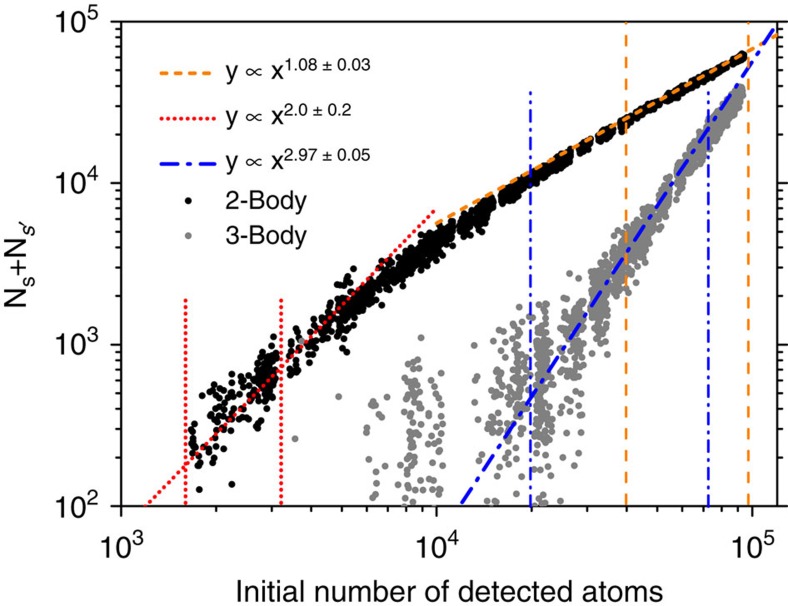
Density dependence. Sum of detected atoms in *s* and 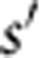 states, 
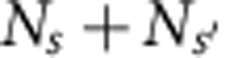
 versus the initial *n*=35 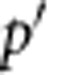 Rydberg atoms density for a transfer time of 
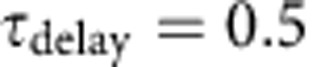
 μs at a fixed electric field of 3.60 V cm^−1^ for the two-body FRET (black dots) in bi-logarithmic scale. Two fits check the quadratic behaviour in the low density regime (red dotted line) and the linear saturation in the high-density regime (orange dashed line). A second set of data are taken at 3.80 V cm^−1^ for the three-body FRET (grey dots) and a fit checks the expected cubic behaviour (blue dotted dashed line). The result and the standard error of each fit is presented in the legend. The data range used for each fit is demarcated by couples of vertical lines with corresponding colour and style.

We vary the Rydberg atom density changing the duration of the Rydberg excitation pulse. Figure 3 presents in a bi-logarithmic scale the resulting number of transferred atoms as a function of the initial number of Rydberg atoms for the two-body FRET at 3.60 V cm^−1^ and for the three-body FRET at 3.80 V cm^−1^. We observe the expected quadratic behaviour of the two-body FRET at the lowest densities but also a fast saturation at moderate densities with a linear dependence once the 50% transfer limit is reached. This saturation occurs at a density about 10 times smaller than our maximum density, that is, ∼5 × 10^8^ atoms cm^−3^ where the coupling *V*_dip_ is around 1.5 MHz at 3-μm distance. This is consistent with the used delay of 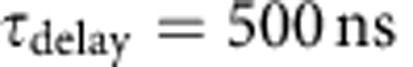. Moreover, the excitation blockade is negligible and cannot explain the saturation at this small density. The measured cubic dependence of the transferred population on the density for the three-body FRET signals confirms our identification of the process. For these measurements with an estimated interaction strength around 3 MHz at the highest density, we observe the beginning of a saturation when reaching the transfer percentage of ∼25%, close to the expected maximum of 33% in this case.

### A general scheme

**Figure 4:**
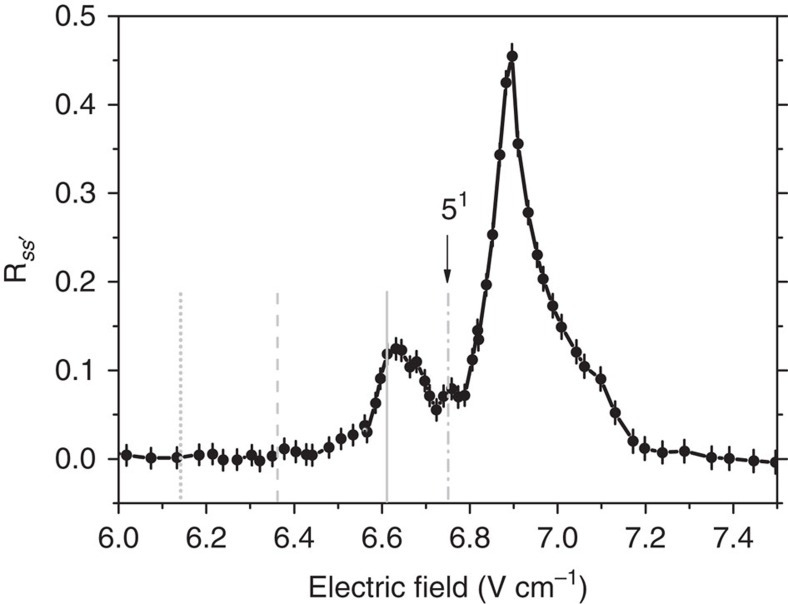
Population transfer from *n*=32. Averaged transfer ratio 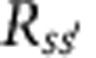 from the initial *p* state to 
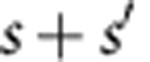
 for *n*=32 with a transfer time of 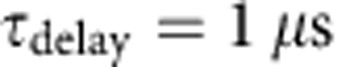. The solid line at 6.61 V cm^−1^ corresponds to the expected field for the three-body FRET. The dashed line at 6.36 V cm^−1^ marks the resonance electric field of the 4^2^ four-body FRET where two relay atoms allow the energy exchange between the last two atoms. The dotted line at 6.14 V cm^−1^ corresponds to the 5^3^ five-body FRET allowed through three relay atoms. Finally, the dashed-dotted line at 6.74 V cm^−1^ corresponds to the 5^1^ five-body FRET where a single relay atom is enough to allow the energy exchange of two pairs of atoms. Each point corresponds to the average of 150–300 individual measurements. The error bars correspond to the sum of the s. e. m. and the estimated error in the state discrimination.

It is important to note here that the idea to use a non-resonant relay atom to compensate for a finite Förster defect is valid for any principal quantum number. For confirmation, we have explored the two-body and three-body FRET's for the *n*=32 Rydberg state at *F*∼6.9 V cm^−1^ and *F*∼6.63 V cm^−1^, respectively, as displayed in Fig. 4. The three-body transfer ratio is about 12% and we have observed a transfer for *n*=28 of about 4%. This rapid decrease of the transfer efficiency is consistent with the *V*_dip_ strong dependence with *n*. These results confirm the general application of few-body FRET processes to any atom, molecule or quantum dot, provided that the relay is able to absorb a small energy mismatch through multiple quasi-degenerate levels or a broad transition line. In Rydberg atoms, it corresponds to any resonance starting from a state with a total angular momentum *J*>1/2.

Our findings also confirm the validity of the many-body diffusion model introduced in ref. 38 to interpret the efficient coupling 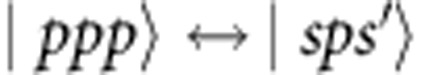 observed through the broadening of the FRET resonance line. That model implies a multistep evolution of the Rydberg excitation transfer, as produced by strong few-body couplings as 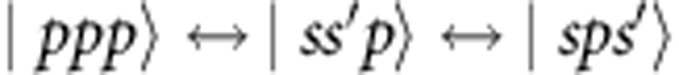 where the *s* or 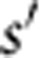 states keep exchanging their energy with surrounding *p* atoms.

## Discussion

In the following discussions, for the sake of simplicity, we will always assume a *p* starting state and the use of the corresponding three-body or few-body resonance electric field. But of course the symmetric situation with the 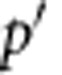 state is equivalent.

As a perspective, we can generalize the few-body FRET further to many-body (Brunnian^39^) FRET:


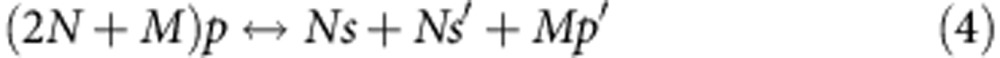


with a resonance condition on the Förster defect defined as:


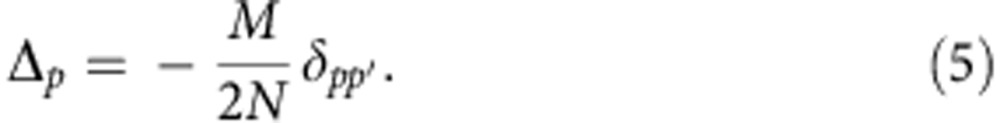


Within this new representation, each resonance can be labelled using the total number of involved atoms 2*N*+*M* and the number of relay atoms *M*, leading to the label (2*N*+*M*)^*M*^. The two-body FRET is thus labelled 2^0^ and the 3-body FRET 3^1^. It is then possible to consider the non trivial 4-body FRET 4^2^, the two 5-body FRET 5^3^ and 5^1^, etc... Fig. 4 highlights with dashed lines the expected resonance fields for 4^2^ at *F*=6.36 V cm^−1^, 5^3^ at *F*=6.14 V cm^−1^ and 5^1^ at *F*=6.74 V cm^−1^. Notice that this last resonance with a very small detuning could contribute to our signal with a peak barely visible out of the noise, although no additional confirmation could be gathered: From equation (5) we find a detuning of *δ*/4 for the 5^1^ resonance, much smaller than the detuning *δ* for the 4^2^ resonance. This could in principle explain why we might observe the first and not the second. Indeed when calculating the interaction strength of these two processes we find 11 kHz for 4^2^ and 25 kHz for 5^1^ at 3 μm. But this last interaction is still too weak to explain the appearence of this peak given the used delay. The clear experimental observation and study of these resonances is a challenge for the next future, demanding improvements in the excitation and detection method, but promising new investigations on the transition from few to many-body physics^38^,^40^.

According to the theoretical study in^33^, the |*ppp*〉 state should present trimer states regardless of the chosen electric field. One can wonder how the additional few-body interactions presented here will modify these trimer states in term of accessibility and stability.

Another prospect of tunable three-body FRET could be found in quantum optics and quantum computing^11^ with the implementation of entanglement or few-body quantum gates. Although the randomness of the Rydberg excitations in our experiment prevented us to demonstrate the coherent nature of the observed three-body FRET, other experiments like the one in ref. 22 realized a precise control of atomic positions. That control should allow interesting applications, as discussed in the following:

The asymmetric role of the relay atom, necessarily ending in the 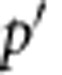 state, forbids *a priori* a three-atom entanglement. But one can in principle symmetrize the problem using a specific atomic arrangement and electric field orientation that garanties equal dipole–dipole interactions between each atom pair. A trivial solution is to use an equilateral triangle with a perpendicular electric field. This idea should still work with 4 atoms, adding the last one out-of-plane at the proper distance giving equal coupling strengths. The implementation of a more general N-body entanglement, for N larger than 4, is much harder to assert without further investigations.

The entanglement of just two particles is already useful in many schemes and the *a priori* knowledge whether the entanglement has been achieved, often called heralded entanglement, is a common way to improve quantum information protocols. Our three-body FRET could provide such an heralded entanglement using the relay atom to report the entanglement of the two others. One can for instance wait for the FRET to take place and ionize the final 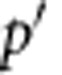 atom after its transfer to a higher state via a resonant microwave field. The detection of this ion will indeed be a proof that the remaining atoms are in the entangled state 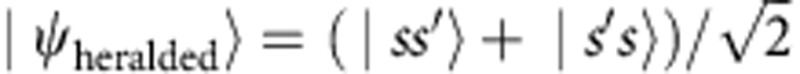.

The ability to implement few-body quantum gates in a single step using few-body interactions might help reducing implementation errors when such gates are required. Evaluating accurately the feasibility and the final error of such gates is beyond the scope of this study but we will present simply one possible scheme. In a linear configuration, mapping the first and last atoms of the chain to the *s* and 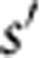 states and the centre atom to either the *p* or 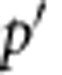 state will implement a controlled Swap gate, also called Fredkin gate. Indeed, if the resonance condition of equation (2) is satisfied, a 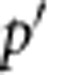 state on the centre atom will lead to the coupling 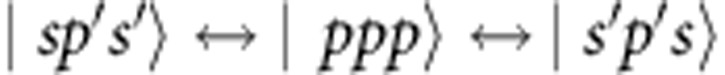 where the *s* and 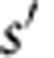 states swap. On the contrary, there is no coupling with a centre *p* atom and the swap doesn't take place.

The resonant energy transfers we have predicted and observed pave the way towards further investigations of the frontier between few-body physics and many-body physics. Their general character, demonstrated in our work, is promising for applications as advanced control techniques in quantum physics.

## Methods

### Rydberg atom source

Our experimental set-up is similar to the one described in ref. 34. We create a caesium magneto-optical trap (MOT) with a typical atomic density of several 10^10^ atoms cm^−3^ and a temperature of ∼100 μK. We then use a three-photon excitation scheme to the Rydberg states *p* or 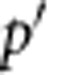. The first photon is provided by the MOT cooling laser, coupling the 6*S*_1/2_ ground state to the 6*P*_3/2_ state at 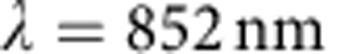. The second step couples the 6*P*_3/2_ to the 7*S*_1/2_ state with a diode laser at 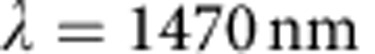. It is switched on for 1 μs with an acousto-optic modulator. The last excitation step couples the 7*S*_1/2_ state to the desired Rydberg state with a cw Ti:Sapphire ring laser at 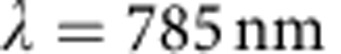. It is switched on for a maximum of 200 ns using another acousto-optic modulator within the previous laser excitation pulse. This duration limit as been chosen to ensure the absence of excitation blockade in the sample, except when the applied electric field is at the two-body resonance. The last two lasers are focused and crossed within the MOT to define a small Rydberg excitation volume of ∼300-μm diameter. With this excitation scheme, we excite up to around 10^5^ Rydberg atoms with a typical Rydberg atom density of ∼5 × 10^9^ atoms cm^−3^.

### Electric field control and Rydberg atom detection

The MOT is located at the centre of four parallel 60 mm by 130-mm wire mesh grids. The centre pair is spaced by 1.88±0.02 cm, while the outer grids are 1.5 cm far from the inner grids. One centre grid is used to apply the required resonance field of several V cm^−1^. The centre grid spacing has been measured observing the *p* state Stark shift as a function of the applied voltage. For the final Rydberg detection, an ionizing field ramp is applied on the other centre grid with an experimental delay 
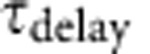
 (300 ns–1 μs) after the end of the Ti:Sa excitation pulse. We apply a voltage of 900 V to ionize the *n*=35 *p* state and 1,400 V to ionize *n*=32. The ions then travel 210 mm to reach a micro-channel plate recording the TOF signal. The different Rydberg states ionize at different fields, thus at different times within the ionization ramp. The ionization field is chosen specifically for each *n* to optimally separate the micro-channel plate signal of *s* and 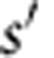 from 
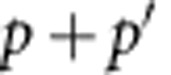
 on the TOF signal. This experimental cycle is then repeated with a 10-Hz repetition rate to accumulate data.

### Data analysis and selection procedure

This detection scheme provides a specific TOF signal for each Rydberg states, but the signals partially overlap and the lowest energy state *s* only partially ionizes. To evaluate accurately the number of atoms in each state, we first measure the TOF signal of the *p*, 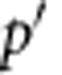, *s* and 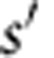 Rydberg states at a non-resonant electric field to serve as TOF references. The reference signals have been measured at an external electric field of 2.70 V cm^−1^ for Fig. 2a and of 4.54 V cm^−1^ for Figs 2b and 3. These TOF references allow us to define optimum time-integration gates and quantify the cross-talks between the various Rydberg signal to account for them^34^. We also measure the ionization fraction of the *s* state and apply a correction factor on this state before processing the transfer ratio. We verify that this correction leads to an equal transfer of *s* and 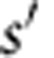 state on the two-body FRET as expected. The residual cross-talk uncertainty is evaluated to ±0.5% maximum between the following channels: *s*, 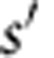 and 
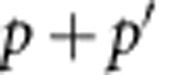
. In our analysis, we choose not to discern between *p* and 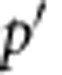 since the two signals are too much overlapped and the residual cross-talk after correction was still on the order of ±5% or more between them. Each reference and each point of the different Fig. 2 corresponds to an average of 50–150 data points selected from 400 to 500 measurements at each value of the electric field. In the case of Fig. 4, we increase the number of measurements up to 900 to save up to 300 data points. This selection is necessary as the Rydberg excitation laser is not frequency locked. The selection is automated in two steps: it first keeps data within ±3 MHz of the optimum excitation frequency while we observe an excitation linewidth of ±5 MHz. It then selects an interval of ±5% in Rydberg atom number containing the largest possible data set. A single interval is used for each of Figs 2 and 4 and an interval is chosen for each excitation pulse duration in Fig. 3. This avoids non-linear interaction effects to play a role within a single point in the figures. After averaging *N* points, the statistical error of the mean (s. e. m.) is estimated by the usual formula of the measured s.d. *σ* divided by 
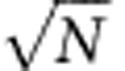
. The error bars in Figs 2 and 4 correspond to the sum of the s.e.m. and the ±0.5% residual cross-talk error. In Fig. 3, we directly plot the whole selected data set for each used value of the Rydberg excitation pulse duration.

## Additional information

**How to cite this article:** Faoro, R. *et al*. Borromean three-body FRET in frozen Rydberg gases. *Nat. Commun*. 6:8173 doi: 10.1038/ncomms9173 (2015).
